# Image quality and artefact reduction for tantalum clips in ocular proton therapy using photon-counting CT

**DOI:** 10.1186/s41747-026-00819-7

**Published:** 2026-11-13

**Authors:** Lea Esser, Christian Bäumer, Benedikt M. Schaarschmidt, Dominik Lisowski, Hannah Steinberg-Vorhoff, Michal-Kamil Chodyla, Aleksandar Milosevic, Beate Timmermann, Jörg Wulff

**Affiliations:** 1 West German Proton Therapy Centre Essen (WPE), Essen, Germany; 2 Department of Physics, TU Dortmund University, Dortmund, Germany; 3 West German Cancer Center (WTZ), University Hospital Essen, Essen, Germany; 4 German Cancer Consortium (DKTK), Essen, Germany; 5 Department of Diagnostic and Interventional Radiology and Neuroradiology, University Hospital Essen, University of Duisburg-Essen, Essen, Germany; 6 Department of Particle Therapy, Essen, Germany; 7 Faculty of Medicine, University of Duisburg-Essen, Essen, Germany; 8 Present Address: Institute of Radiology, Neuroradiology and Interventional Therapy, University of Erlangen-Nuremberg, Medical Campus Upper Franconia, Klinikum Bayreuth, Bayreuth, Germany

**Keywords:** Artefacts, Image enhancement, Proton therapy, Tantalum, Tomography

## Abstract

**Objective:**

Tantalum fiducials (“clips”) used in intraocular proton therapy generate strong artefacts in conventional computed tomography, limiting the visibility of tumours and anatomical structures. This study investigated the potential of photon-counting computed tomography (PCCT) for reducing tantalum-related artefacts.

**Materials and methods:**

Two phantom models containing tantalum clips were examined using a photon-counting CT system and two conventional CT scanners with energy-integrating detectors (EICT). Reconstruction parameters, including tube voltage, image resolution, reconstruction kernel, virtual monoenergetic imaging (VMI), and iterative metal artefact reduction (iMAR), were systematically optimised. Image quality was evaluated using objective metrics assessing homogeneity, noise, contrast, structural similarity, and artefact volume, complemented by a blinded subjective observer study. Correlations between objective and subjective results were analysed using Spearman’s rank correlation.

**Results:**

Objectively, PCCT showed no significant advantage over the modern EICT scanner but significantly outperformed the older-generation EICT therapy simulator. In contrast, blinded observers consistently preferred PCCT images. iMAR was the dominant factor influencing image quality. VMI alone did not yield relevant improvements. High-energy VMI (110 keV) combined with iMAR achieved the best subjective ratings. Higher spatial resolution due to smaller voxel sizes showed no clinically relevant benefit for artefact reduction. Soft reconstruction kernels provided the best balance between artefact suppression and image noise. Objective rankings correlated strongly with subjective assessments.

**Conclusion:**

PCCT combined with iMAR improves subjective image quality in intraocular proton therapy and reduces the perceived severity of tantalum artefacts. Nevertheless, tantalum remains a major challenge in image-guided proton therapy, requiring further optimisation of imaging and reconstruction strategies.

**Key Points:**

***Question*** Photon-counting CT may improve visualisation of tantalum clips and reduce metal artefacts in ocular proton therapy planning compared with conventional CT systems.

***Findings*** Photon-counting CT achieved the highest subjective image quality ratings, while iterative metal artefact reduction remained the dominant factor influencing artefact suppression.

***Relevance statement*** Photon-counting CT combined with iterative metal artefact reduction can improve visualisation of tantalum clips and surrounding anatomy in ocular proton therapy planning and may support more reliable tumour localisation despite limited objective differences compared with state-of-the-art conventional CT.

**Graphical Abstract:**

**Figure:**
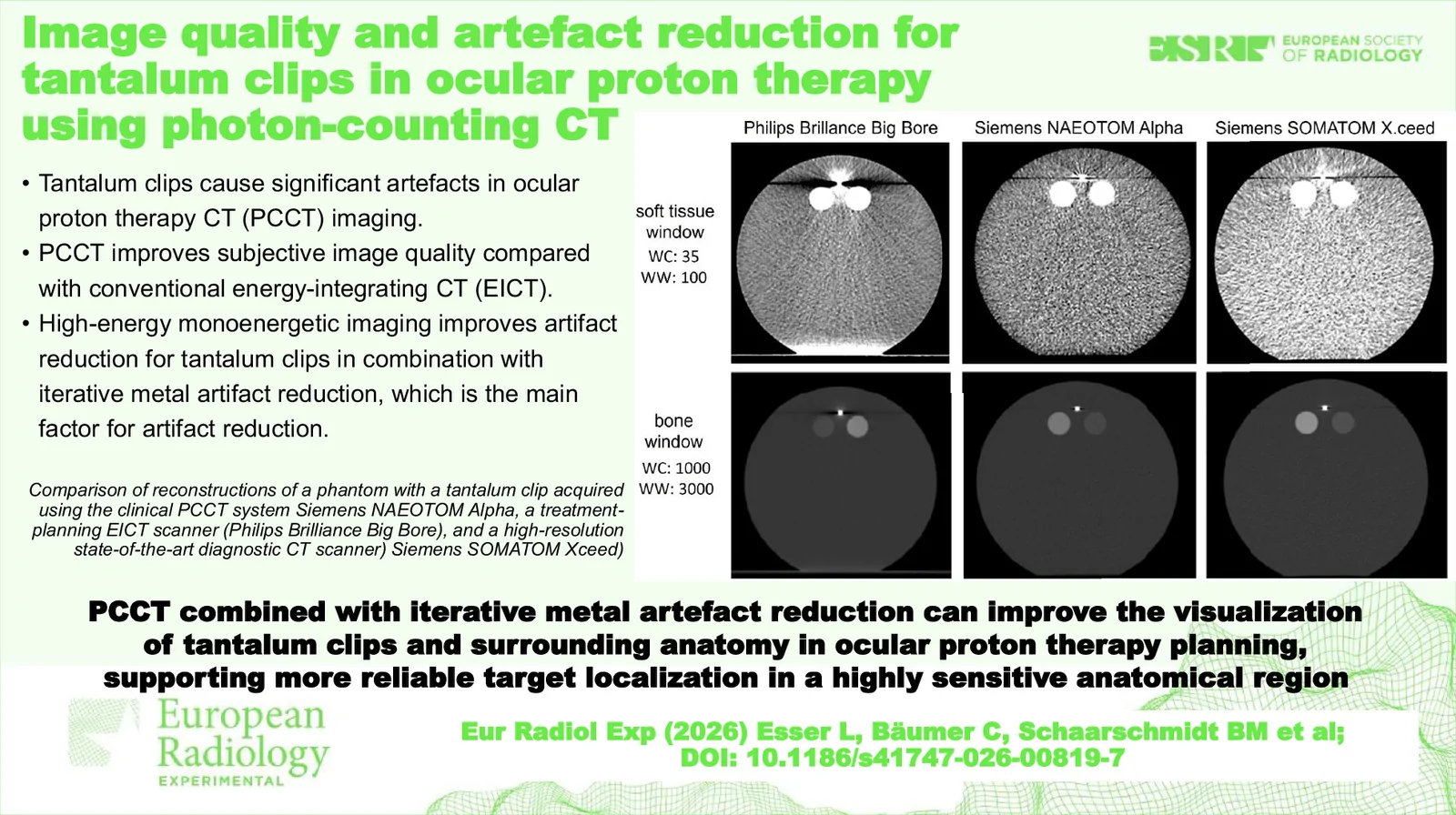

## Background

Uveal melanoma is the most common adult primary intraocular malignancy. Ocular proton therapy (OPT) achieves > 95% local tumour control, high eye preservation, and functional vision retention [1–3]. Traditionally, OPT treatment planning relies on a simplified eye model from orthogonal x-ray images, using tantalum fiducial markers (“clips”) sutured to the sclera to allow reconstruction of a 3D eye and tumour model as well as position verification during radiotherapy [4, 5]. x-ray computed tomography (CT) has been incorporated into the OPT workflow [6], but tantalum causes extensive artefacts, often obscuring the lesion, and the limited spatial resolution of conventional CT restricts detailed visualisation of ocular structures.

Photon-counting CT (PCCT) offers a potential solution to these limitations *via* energy-resolved photon detection, submillimetre spatial resolution, improved contrast-to-noise ratio, and spectral imaging capabilities [7]. Several studies demonstrated reduced metal artefacts using PCCT, particularly through virtual monoenergetic imaging (VMI) above tantalum’s K-edge [8–13]. For conventional energy-integrating CT (EICT), image quality near metals can be improved with iterative metal artefact reduction (iMAR) [14], increased tube voltage [15], tin filtration [8, 11], and kernel selection [16]. Against this background, this study systematically investigates PCCT’s potential compared to conventional EICT systems for OPT.

## Materials and methods

### Scanners and phantoms

A clinical PCCT system (NAEOTOM Alpha, Siemens Healthineers, Forchheim, Germany) was compared with two EICT systems: a treatment planning (TP) scanner (Brilliance Big Bore, Philips Healthcare, Cleveland, OH, USA) and a high-resolution state-of-the-art (SOTA) diagnostic CT scanner (SOMATOM X.ceed, Siemens Healthineers, Forchheim, Germany). Acquisition parameters were matched across CT systems where technically feasible, using protocol-specific adjustments (*e.g*., Sn 140 kV) to maintain comparable image quality and reflect clinical ocular proton therapy workflows. Radiation exposure was defined by each clinical protocol and was not dose-matched.

A range of acquisition and reconstruction parameters was evaluated, including iMAR, tube voltages of 120 kV and tin-filtered (Sn) 140 kV, and two in-plane resolution settings: high-resolution (HR) and ultra-high-resolution (UHR) mode. Matrices of 512² (field of view (FOV) = 20 × 20 cm²) and 1,024² (FOV = 9 × 9 cm²) were combined with soft, medium, and hard reconstruction kernels (Qr/Hr36, Qr/Hr40, and Qr/Hr56). VMIs were analysed at energy levels of 65 keV, 70 keV, 80 keV, and 110 keV combined with iMAR for the PCCT system.

Results of a subjective and objective evaluation of the PCCT were compared with the EICT systems with similar CT reconstructions (see Table 1).

**Table 1:** CT reconstructions used for subjective image quality assessment

Reconstruction name (CT, U, CT mode, kernel, iMAR)	CT	U/kV	iMAR	Voxel size: CT mode, matrix size, FOV size	Kernel	Effective Slice-thickness*/mm
N, Sn 140, HR, Qr40, df	Siemens NAEOTOM Alpha	Sn 140	Dental fillings	HR, 512^2^, 20 × 20 cm^2^	Qr40	0.2
N, 120, HR, Qr40, none	Siemens NAEOTOM Alpha	120	None	HR, 512^2^, 20 × 20 cm^2^	Qr40	0.2
N, 120, HR, Qr40, df	Siemens NAEOTOM Alpha	120	Dental fillings	HR, 512^2^, 20 × 20 cm^2^	Qr40	0.2
N, VMI 110, HR, Qr40, df	Siemens NAEOTOM Alpha	VMI 110 keV	Dental fillings	HR, 512^2^, 20 × 20 cm^2^	Qr40	0.2
N, 120, HR, Qr40, nc	Siemens NAEOTOM Alpha	120	Neuro coils	HR, 512^2^, 20 × 20 cm^2^	Qr40	0.2
N, 120, HR, Qr36, df	Siemens NAEOTOM Alpha	120	Dental fillings	HR, 1,024^2^, 20 × 20 cm^2^	Qr36	0.2
N, 120, HR, Qr56, df	Siemens NAEOTOM Alpha	120	Dental fillings	HR, 1,024^2^, 20 × 20 cm^2^	Qr56	0.2
N, 120, UHR, Qr40, df	Siemens NAEOTOM Alpha	120	Dental fillings	UHR, 1,024^2^, 9 × 9 cm^2^	Qr40	0.1
P, 120, HR, UB, none	Philips Brilliance Big Bore	120	None	HR, 512^2^, 13.5 × 13.5 cm^2^	UB	0.325
X, 120, HR, Hr40, df	Siemens SOMATOM X.ceed	120	Dental fillings	HR, 1,024^2^, 20 × 20 cm^2^	Hr40	0.3
X, 120, HR, Hr40, nc	Siemens SOMATOM X.ceed	120	Neuro coils	HR, 1,024^2^, 20 × 20 cm^2^	Hr40	0.3

*CT* Computer tomography, *df* Dental fillings, *FOV* Field of view, *HR* High-resolution mode, *Hr/Qr/UB* Manufacturer-specific reconstruction kernels, *iMAR* Iterative metal artefact reduction, *N* Siemens NAEOTOM Alpha, *nc* Neuro coils, *P* Philips Brilliance Big Bore, *Sn* Tin filtration, *UHR* Ultra-high-resolution mode, *VMI* Virtual monoenergetic imaging, *X* Siemens SOMATOM X.ceed

The iMAR options “dental fillings” and “neuro coils”, as well as the other optimised reconstruction parameters, were applied on the Siemens scanners, whereas the clinical OPT protocol of the Philips EICT system was used without additional metal artefact reduction (*e.g*., OMAR).

### Phantoms

**Fig. 1:**
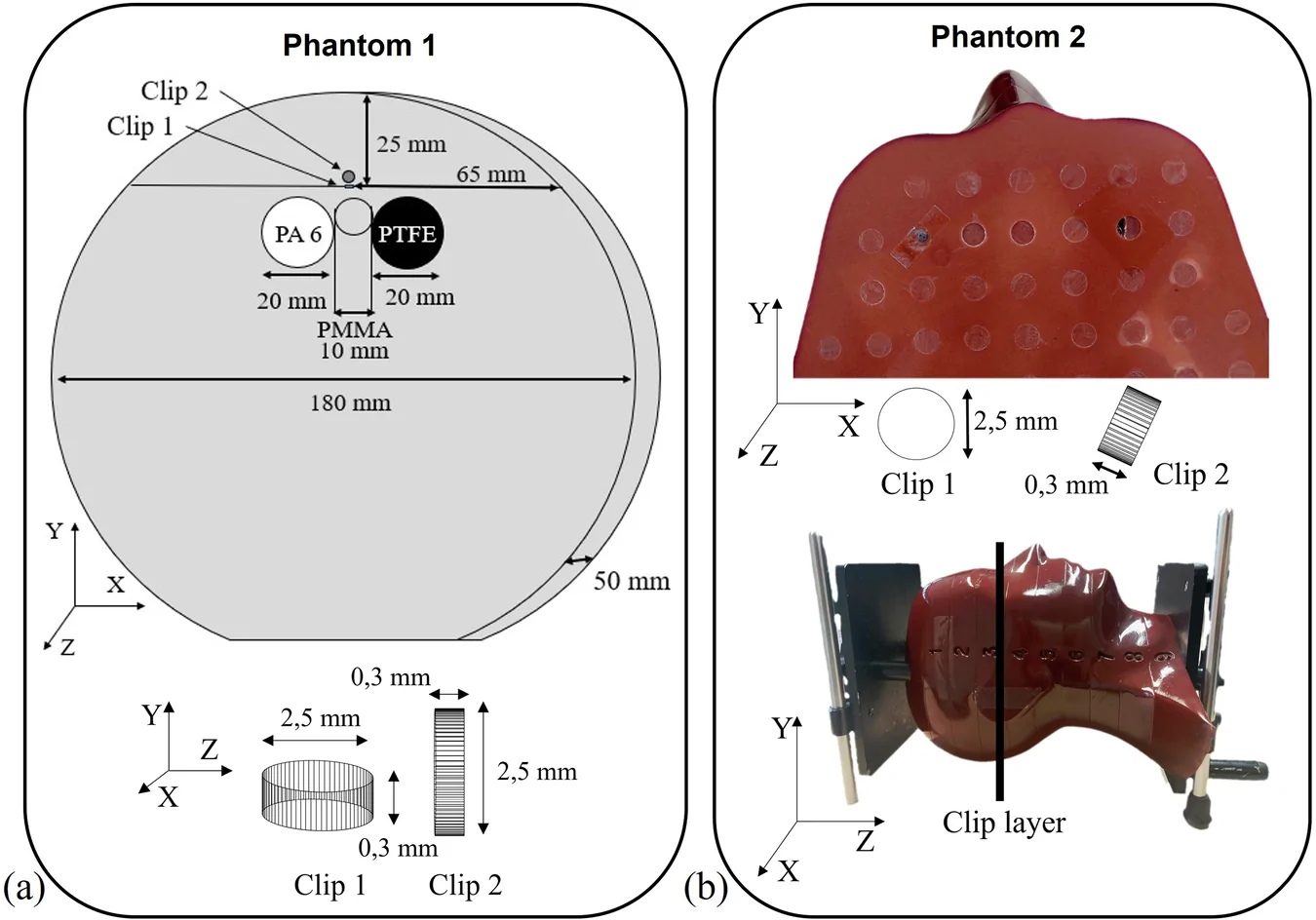
Schematic representation of (**a**) PMMA phantom (Phantom 1) and (**b**) head phantom (Phantom 2) with clips and inserts. Dimensions and orientations from different views. For details, see text. PA6, Polyamide 6; PMMA, Polymethylmethacrylate

Two phantoms containing 2.5 mm diameter tantalum clips with 0.3 mm thickness (Altomed Limited, Boldon, England) were imaged (Fig. 1).

Phantom 1, a bevelled polymethylmethacrylate (PMMA) cylinder, contained clips in three defined orientations for assessing artefacts: horizontally (with the base parallel to the CT Z-axis), vertically (perpendicular to the Z-axis), and obliquely, achieved by mounting the Phantom 1 on a 45° foam wedge.

Three cylindrical inserts were embedded below the clips to assess contrast detectability, composed of polyamide 6 (PA6, mass density *ρ*_PA6_ = 1.19 g/cm^3^, radius *r* = 5 mm), PMMA (*ρ*_PMMA_ = 1.14 g/cm^3^, *r* = 10 mm), and polytetrafluorethylene (PTFE, *ρ*_PTFE_ = 2.16 g/cm^3^, *r* = 10 mm). Densities were chosen to represent relevant contrast: the difference between PA6 and PMMA (0.05 g/cm^3^) simulates the contrast between soft tissues in the orbit, while the difference between PTFE and PMMA (1.02 g/cm^3^) approximates the contrast between soft tissue and cortical bone [17].

Phantom 2, an anthropomorphic head phantom (Alderson-Rando phantom head, Radiology Support Devices, Inc. (RSD), Long Beach, CA, USA), was equipped with a tantalum clip at the level of each orbit, with the base perpendicular to the Z-axis, and the other in an oblique orientation within an air-filled recess.

### Objective image evaluation

**Fig. 2:**
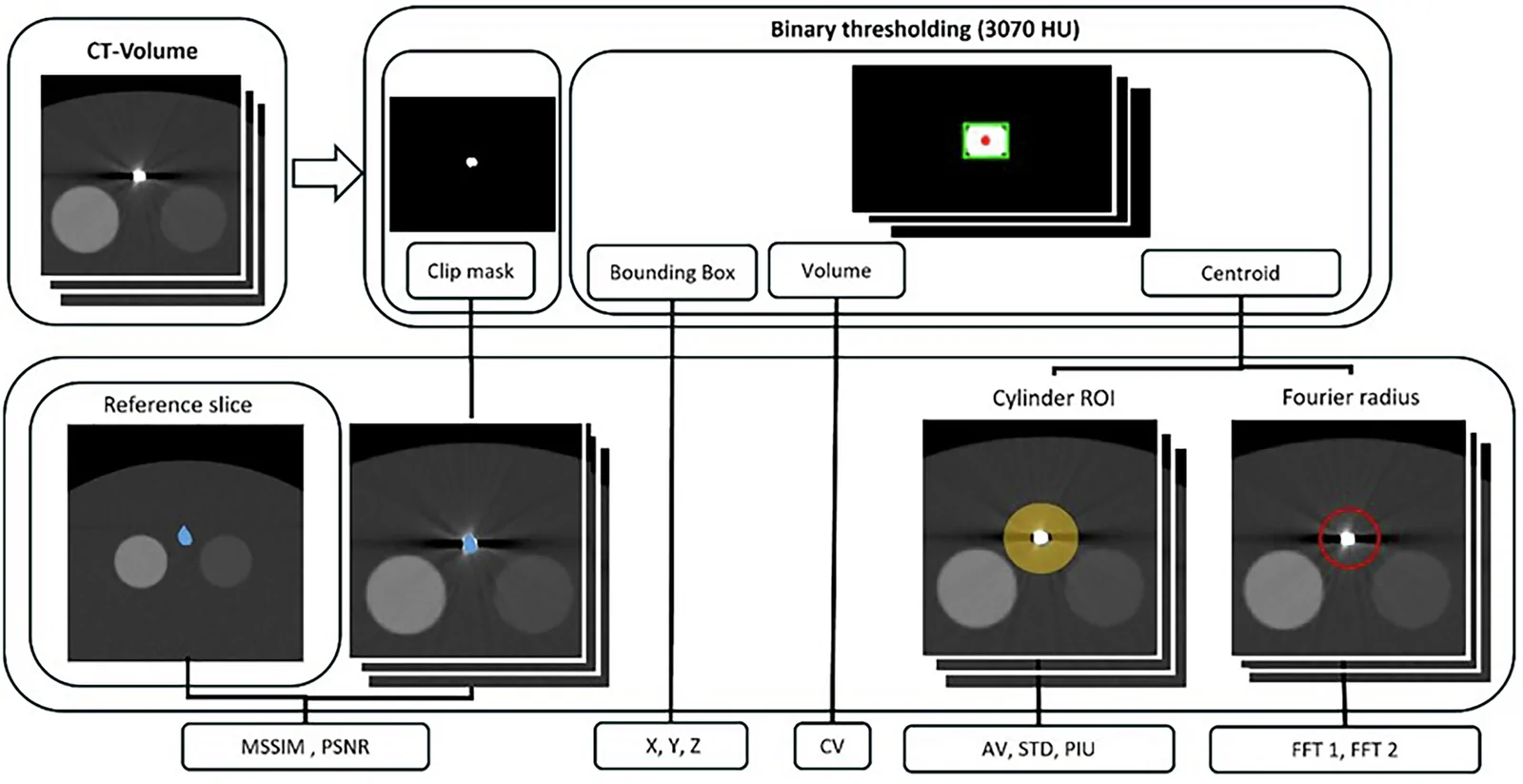
Schematic flow of objective image evaluation. For details, see text. AV, Artefact volume; CT, Computer tomography; CV, Clip volume; FFT 1, Mean maximum amplitude of the low-frequency angular fast Fourier transform spectrum; FFT 2, Mean integrated amplitude of the low-frequency fast Fourier transform spectrum; HU, Hounsfield units; MSSIM, Mean structural similarity index; PIU, Percentage intensity uniformity; PSNR, Peak signal-to-noise ratio; ROI, Region of interest; STD, Standard deviation; X/Y/Z, Clip widths

An automated MATLAB R2025a (The Mathworks, Massachusetts, USA) pipeline was implemented for analysis and calculation of image quality metrics (Fig. 2).

Clips were segmented at a 3,070 Hounsfield units (HU) threshold, motivated by the upper limit of 3,071 HU for CT scanners with 12-bit grey value representation. The centroid, bounding box (smallest fully enclosing cuboid) with dimensions X/Y/Z, and clip volume (CV) of this mask were determined. The CV was compared to the clips’ true volume to assess reconstruction accuracy.

Image quality was assessed *via* peak signal-to-noise ratio (PSNR) and mean structural similarity index (MSSIM) [18–22], by comparing each CT slice with a reference slice without clip, excluding the clip mask region.

A cylindrical region of interest (ROI) at the binary mask centroid (inner radius = 2 mm, outer radius = 7.5 mm) determined mean, minimum HU_min_, maximum HU_max_, and standard deviation (STD), the latter as a measure of image noise due to artefacts [9, 11, 23, 24]. The artefact volume (AV) was determined as the number of voxels within the ROI with HU > 3*standard deviations of mean, calculated in the reference slice (without clip). Following Große-Hokamp et al [25], percentage intensity uniformity (PIU) was calculated representing homogeneity within the cylinder ROI.

Finally, a frequency-based analysis using a fast Fourier transform (FFT), following the approach of Große-Hokamp et al [25], quantified streak artefacts. A circular ROI with 4 mm radius (“Fourier radius” in Fig. 1) was defined around the centroid of each clip. The maximum and integrated amplitude in the range 0/° to 10^-1^/° was calculated per slice, capturing low-frequency components of streaks, while excluding higher-frequencies associated with noise. The mean values of the maximum amplitude (FFT 1) and the integrated amplitude (FFT2) were calculated in the clip mask along the Z direction.

### Evaluation scheme based on objective evaluation metrics

To identify the most effective reduction in artefacts, a ranking for the above-described metrics was applied by comparing each metric to a target value ${T}_{{j}}$ and assigning a weighting for the overall ranking. The deviation ${A}_{i,{j}}$ from the target value ${T}_{{j}}$ was calculated with

$${A}_{i,j}=|{T}_{j}-{x}_{i,j}|,$$

where ${x}_{i,{j}}$ is the determined value for metric j and reconstruction i. By ordering ${A}_{i,{j}}$ for each reconstruction, a rank starting at 0 was defined, averaged in case of a tie. By further normalising over the number of available N reconstructions, a rank value ${r}_{i,j}$ between 0 and 1 resulted, allowing to compare different scanners and reconstructions.

A weighted rank sum ${S}_{i,k}$ was calculated with weights ${w}_{j}$ and ${r}_{i,j}$ per clip k with

$${S}_{i,k}={\sum }_{j=1}^{m}{r}_{i,j}\cdot {w}_{j}.$$

The evaluation was performed separately for clips 1 and 2 of each scan. The normalised ranks of both clips were averaged to give the final overall rank.

Clip dimensions X/Y/Z along the corresponding axes and CV were considered to equally reflect clip reconstruction accuracy with ${w}_{j}$ = 0.25. The target values ${T}_{{j}}$ corresponded to the actual dimensions and volume of the clip. AV, PIU, MSSIM, and PSNR were considered with ${w}_{j}$ = 1, and metrics FFT 1 and FFT 2 were weighted equally with ${w}_{j}$ = 0.5. Depending on the metric, the target value ${T}_{{j}}$ was either zero (AV, FFT 1 & 2) or 1 (PIU, MSSIM, PSNR).

### Evaluation of contrast in the periphery of tantalum clips

To assess contrast detectability near the clips, contrast-to-noise ratio (CNR) of the cylindrical inserts in Phantom 1 was calculated following Baldelli et al [26]. Mean HU values and STD were computed within a cylindrical ROI across the full clip extent in Z (defined by the bounding box). The ROI radius was set to 80% of the insert diameter (8 mm). A reference ROI (*r* = 15 mm) was positioned at the artefact-free periphery of the phantom.

### Subjective image evaluation

Four radiologists (5–10+ years of experience) and one radiation oncologist rated phantom images on a 6-point Likert scale. Evaluation included overall image quality in relation to metal artefact reduction (MAR, 1 = very poor image quality with insufficient MAR; 6 = very good image quality with effective MAR), clip delineation (1 = very strong artefacts, clip size cannot be accurately estimated; 6 = minimal artefacts, clip size can be estimated accurately), artefact severity (1 = very strong artefacts; 6 = minimal artefacts), and the impact of artefacts on the visibility of nearby and distant structures in terms of homogeneity and contrast (1 = very strong impact; 6 = no impact).

Participants evaluated 44 CT images (eleven reconstructions of two phantoms in two window settings). Axial images at Clip 1 (centre of mass) for Phantom 1 and at the midpoint between centroids of both clips for Phantom 2 were selected. Both images were presented in soft tissue as well as bone window/level settings, *i.e*., window centre (WC) and width (WW) for soft tissue at 130/100 HU (Phantom 1) and 35/100 HU (Phantom 2) and for bone 1,000/3,000 HU (both phantoms). Reconstructions were selected to represent key parameter variations of the PCCT and the two EICT scanners (see Table 1). Participants were informed of the phantom type but blinded to scanner and reconstruction parameters to ensure an unbiased assessment.

### Correlation of objective and subjective image evaluation

The correlation and agreement between objective rating and subjective image evaluation were assessed with Spearman’s rank correlation coefficient (ρ). A reliable conclusion was considered for ρ > 0.9 (95% confidence interval) for the sample size of 11 reconstructions [27]. In addition, statistical significance was calculated for correlation across all reconstructions (${p}_{{total}}$) and per phantom (${p}_{{phantom}}$). Following common statistical convention, p < 0.05 was considered significant [28].

## Results

### Objective evaluation of various PCCT parameters

#### Impact of iterative metal artefact reduction (iMAR)

**Fig. 3:**
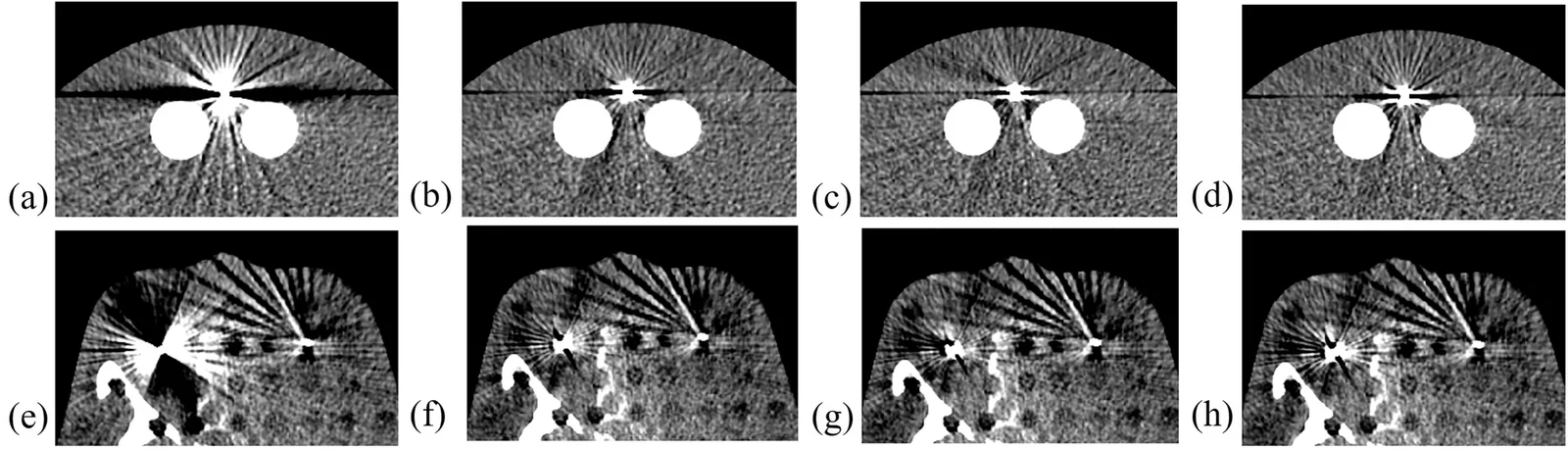
Comparison of PCCT axial images using different iMAR algorithms: (**a**, **e**) without iMAR, (**b**, **f**) “neuro coils”, (**c**, **g**) “dental fillings”, and (**d**, **h**) “extremity implants” for Phantom 1 ((**a**–**d**), WC: 130 HU, WW: 100 HU) and Phantom 2 ((**e**–**h**), WC: 35 HU, WW: 100 HU). iMAR, Iterative metal artefact reduction; PCCT, Photon-counting computer tomography; WC, Window centre; WW, Window width

Figure 3 shows reconstructions with the three best-ranked iMAR algorithms (“dental fillings”, “neuro coils” and “extremity implants”) and a reconstruction without iMAR.

“Neuro coils” iMAR ranked highest across both phantoms, keeping clip diameter deviations generally < 1 mm with, except for Clip 1 in Phantom 1 with a deviation of 2.4 mm in the y-direction. Figure S1 shows the deviations of the single evaluation metrics for the three best-ranked iMAR algorithms compared to reconstructions without iMAR.

Evaluation of contrast detectability revealed “neuro coils” showing a lower contrast-to-noise ratio (CNR) in low contrast compared to images without iMAR, while “extremity implants” and “dental fillings” achieved a higher CNR in high and low contrast. Given the clinical importance of low-contrast detectability in ophthalmologic imaging, “dental fillings” was selected for further analysis.

#### Impact of virtual monoenergetic image reconstruction (VMI)

VMI alone (65–110 keV) did not substantially reduce metal artefacts. VMI at 70 keV yielded the most favourable balance between image PIU, MSSIM, and CNR (see Tables S1 and S2). No substantial improvement in artefact-related parameters (AV, FFT 1/2) was observed at any energy level.

**Fig. 4:**
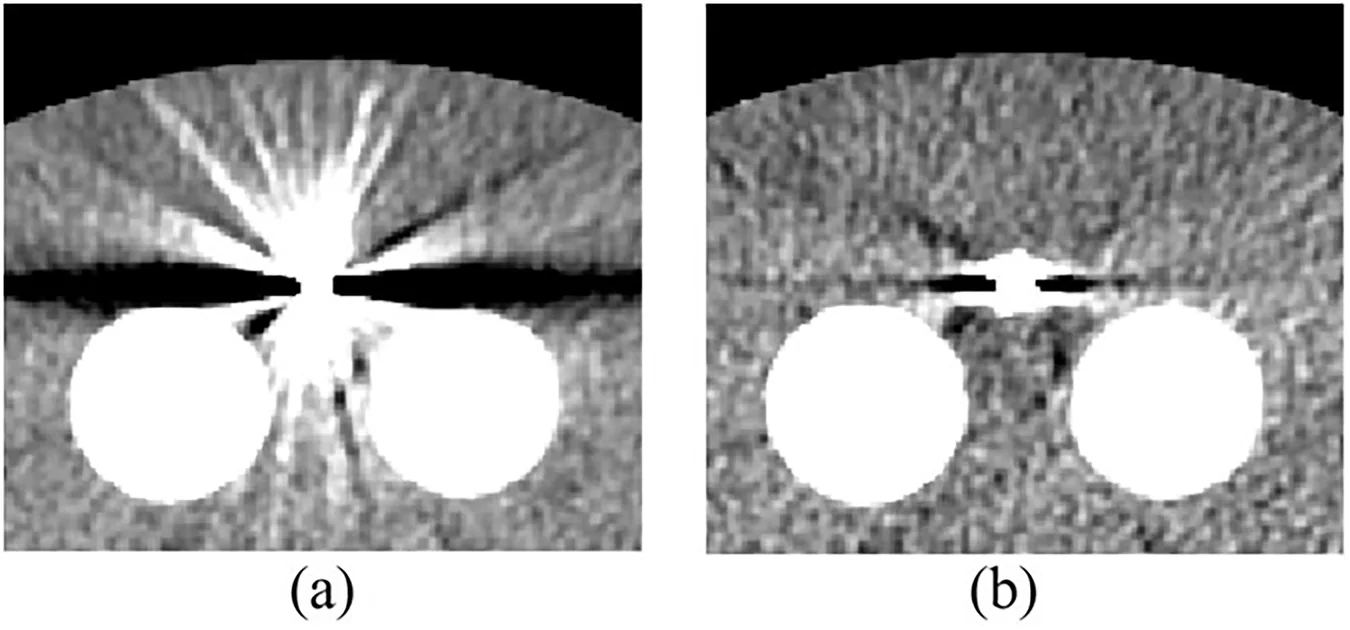
Comparison of the VMI at 110 keV: (**a**) without iMAR and (**b**) with iMAR dental fillings of the Siemens NAEOTOM Alpha (WC: 148 HU, WW: 100 HU). iMAR, Iterative metal artefact reduction; VMI, Virtual monoenergetic imaging; WC, Window centre; WW, Window width

By combining high-energy VMI at 110 keV with iMAR, an effective reduction in metal artefacts was achieved (see example in Fig. 4), with “neuro coils” showing overall the highest-ranked objective result (Tables S1 and S2).

#### Additional parameter optimisation

In the standard orbital CT protocol [29], a tube voltage of 120 kV is routinely applied. A higher tube voltage in combination with Sn filter did not improve ranking (Tables S1 and S2).

The UHR mode (9 × 9 cm²) yielded the lowest objective ranking. Although the finer voxel size improved geometric delineation in the z-axis direction, it markedly increased noise and artefact appearance, likely due to reduced partial-volume averaging. As a consequence, small intensity fluctuations increased, resulting in larger AV and STD within the ROIs, while CNR decreased for high/low-contrast inserts. The HR mode (20 × 20 cm²) offered the most favourable compromise between geometric accuracy for reconstructed single clips (with diameter deviations of < 1 mm) and quantitative metrics (AV, STD, CNR). Therefore, increasing the in-plane resolution beyond the HR setting did not enhance tantalum artefact reduction.

The reconstruction kernel had a pronounced impact on quantitative metrics for noise and artefact representation. Soft kernels (Qr36) produced higher CNR and more consistent clip diameters, whereas hard kernels (Qr56) reduced AV by edge enhancement, but increased image noise, indicated by elevated STD values.

Finally, clip orientation affected the severity of artefacts. A perpendicular alignment of the clip base to the CT z-axis minimised dimensional deviations (< 1 mm) and reduced hyperdense streaks. Oblique or z-parallel orientations produced stronger peripheral artefacts and diameter deviations up to 2.4 mm, confirming that orthogonal positioning provides the most favourable geometry for artefact suppression and dimensional accuracy.

A complete overview of all quantitative results is provided in Tables S1 and S2.

#### Comparison of PCCT with EICT systems

**Fig. 5:**
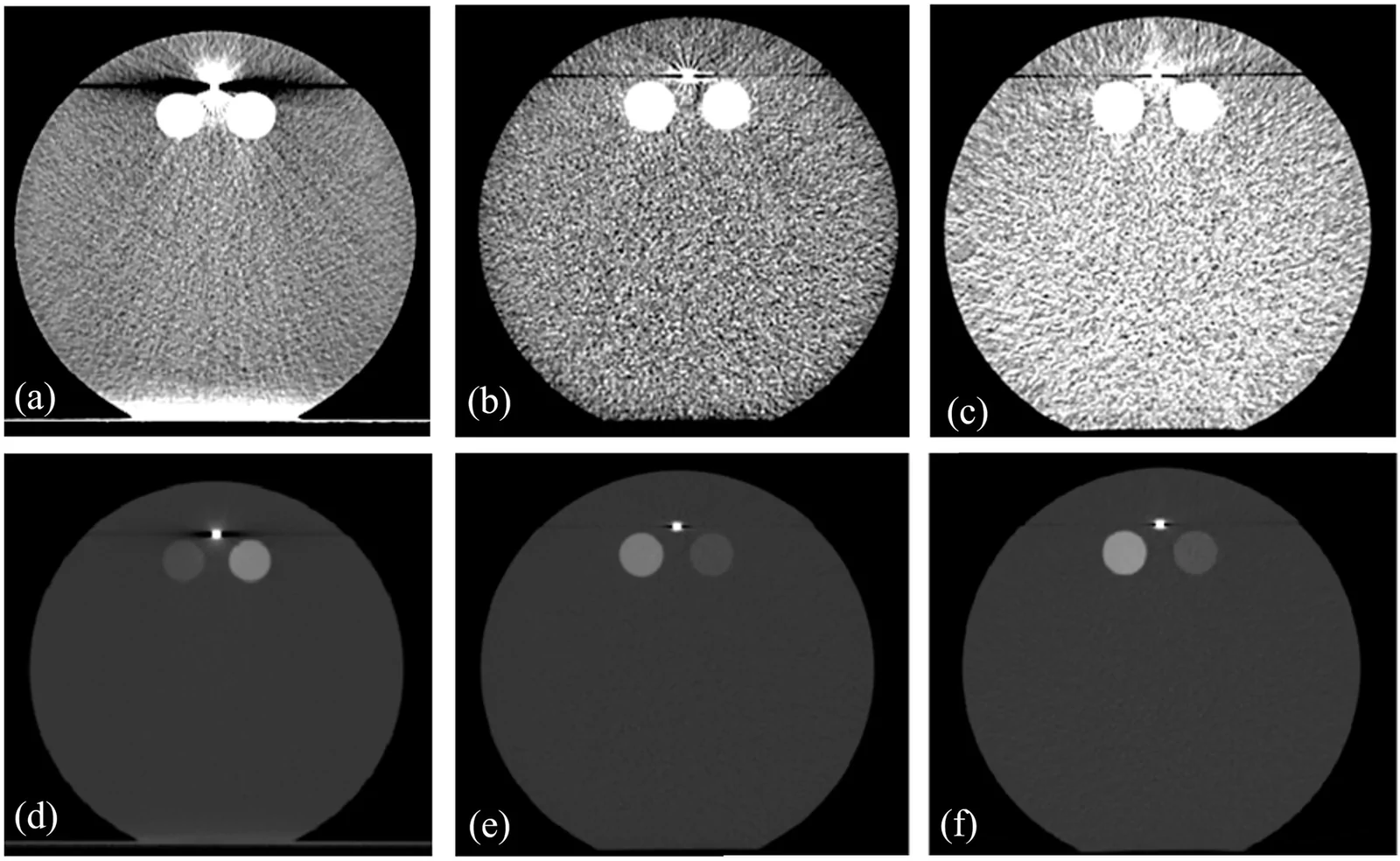
Comparison of CT reconstructions of the (**a**, **d**) PCCT Siemens NAEOTOM Alpha (N, 120, HR, Qr40, df) with (**b**, **e**) EICT Siemens SOMATOM X.ceed (X, 120, HR, Hr40, df) and (**c**, **f**) clinical protocol of EICT Philips Brilliance Big Bore (P, 120, HR, UB, none). Phantom 1 Clip 1 is shown as an example in soft tissue (**a**–**c**) and bone window (**d**–**f**) (for reconstruction parameters, see Table 1. df, Dental fillings; EICT, Energy-integrating computer tomography; HR, High-resolution mode; Hr/Qr/UB, Manufacturer-specific reconstruction kernels; N, Siemens NAEOTOM Alpha; P, Philips Brilliance Big Bore; PCCT, Photon-counting computer tomography; X, Siemens SOMATOM X.ceed

Reconstructions for the three investigated scanners are shown exemplarily in Fig. 5.

**Fig. 6:**
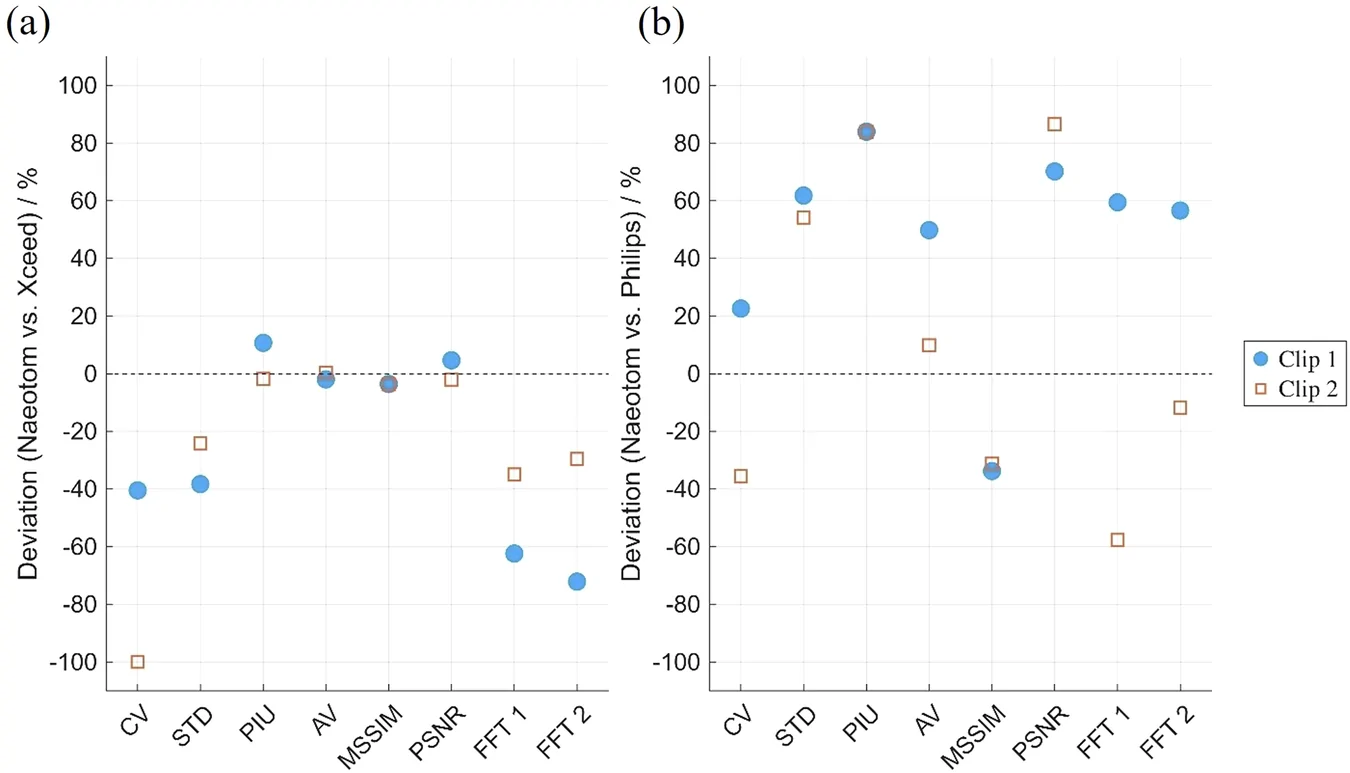
**a** Percentage deviation of image quality metrics of the PCCT NAEOTOM Alpha (Naeotom) relative to the EICT SOMATOM X.ceed (Xceed), using iMAR dental fillings (U: 120 kV, kernel: Qr40/Hr40, CT mode: HR, matrix size: 1,024², FOV: 20 × 20 cm²). **b** Percentage deviation of PCCT relative to EICT (Philips). Positive values indicate improvement and negative values indicate a degradation of the respective metric for the PCCT to EICT system. Deviation < -100% are truncated at -100% (original value -716%). AV, Artefact volume; CV, Clip volume; EICT, Energy-integrating computer tomography; FFT 1, Mean maximum amplitude of the low-frequency angular fast Fourier transform spectrum; FFT 2, Mean integrated amplitude of the low-frequency fast Fourier transform spectrum; FOV, Field of view; HR, High-resolution mode; iMAR, Iterative metal artefact reduction; MSSIM, Mean structural similarity index; Naeotom, Siemens NAEOTOM Alpha; PCCT, Photon-counting computer tomography; PIU, Percentage intensity uniformity; PSNR, Peak signal-to-noise ratio; STD, Standard deviation; Xceed, Siemens SOMATOM X.ceed

A direct comparison based on the objective ranking scheme is summarised in Table 2. PCCT with iMAR outperformed TP EICT, particularly for the parameters describing AV, PIU and STD. However, no significant advantage over SOTA EICT was observed. For illustration, the percentage deviation in metric scores between the PCCT and the SOTA EICT (same reconstruction parameters) is shown in Fig. 6. The percentage deviations of the PIU, AV, MSSIM and PSNR between both Siemens systems remained small, while CV, STD, as well as FFT 1 and 2, were even less favourable for PCCT.

**Table 2:** Rank-based evaluation of objective image quality evaluation, based on Clip 1 (Phantom 1) and mean of Clip 1/2 (Phantom 2) to match subjective analysis

Reconstruction	Normalised ranks											Normalised sum of ranks	Rank Clip	Final Rank
	X	Y	Z	CV	AV	PIU	STD	MSSIM	PSNR	FFT 1	FFT 2			
**Phantom 1**														
X, 120, HR, Hr40, df	0.00	0.00	0.35	0.00	0.00	0.60	0.20	0.30	0.60	0.00	0.00	0.00	1	1
X, 120, HR, Hr40, nc	0.00	0.00	0.35	0.10	0.10	0.80	0.70	0.40	0.70	0.20	0.50	0.35	5	2
N, 120, HR, Qr36, df	0.37	0.42	0.35	0.50	0.80	0.30	0.00	0.00	0.40	0.30	0.20	0.09	3	3
N, VMI 110, HR, Qr40, df	0.68	0.42	0.10	0.80	0.30	0.00	0.30	0.50	0.10	0.70	0.10	0.08	2	4
N, 120, HR, Qr40, nc	0.68	0.42	0.75	0.60	0.50	0.40	0.50	0.70	0.20	0.40	0.60	0.41	7	5
N, 120, HR, Qr40, df	0.68	0.42	0.75	0.30	0.70	0.20	0.10	0.80	0.30	0.10	0.30	0.26	4	6
N, Sn 140, HR, Qr40, df	0.68	0.42	0.35	0.40	0.40	0.10	0.40	0.90	0.50	0.50	0.40	0.36	6	7
N, 120, HR, Qr56, df	1.00	0.89	0.75	0.90	0.20	0.90	0.80	1.00	0.00	0.80	0.80	0.70	9	8
N, 120, UHR, Qr40, df	0.26	0.79	0.00	0.20	0.60	0.50	0.60	0.20	0.90	0.60	0.70	0.50	8	9
P, 120, HR, UB, none	0.16	1.00	1.00	1.00	1.00	1.00	0.90	0.10	1.00	1.00	0.90	1.00	11	10
N, 120, HR, Qr40, none	0.68	0.42	0.75	0.70	0.90	0.70	1.00	0.60	0.80	0.90	1.00	0.96	10	11
**Phantom 2**														
N, VMI 110, HR, Qr40, df	0.65	0.96	0.91	0.83	0.20	0.42	0.28	0.26	0.10	0.00	0.00	0.03	1.5	1
X, 120, HR, Hr40, df	0.00	0.09	0.00	0.00	0.35	0.00	0.00	0.37	0.60	0.50	0.47	0.00	2	2
X, 120, HR, Hr40, nc	0.00	0.00	0.00	0.00	0.30	0.47	0.33	0.26	0.80	0.78	0.58	0.26	5	3
N, Sn 140, HR, Qr40, df	0.50	0.96	0.91	0.39	0.65	0.21	0.22	0.84	0.20	0.06	0.05	0.23	4.5	4
N, 120, HR, Qr40, nc	0.88	0.96	0.46	0.83	0.40	0.26	0.33	0.58	0.50	0.28	0.32	0.30	5.5	5
N, 120, HR, Qr36, df	0.38	0.48	0.77	0.33	0.80	0.21	0.28	0.00	0.80	0.50	0.47	0.30	6	6
N, 120, UHR, Qr40, df	1.00	1.00	0.17	0.17	0.25	0.63	0.61	0.53	0.30	0.22	0.42	0.31	5.5	7
N, 120, HR, Qr40, df	0.65	0.96	0.46	0.61	0.65	0.32	0.33	0.68	0.50	0.17	0.21	0.35	6.5	8
N, 120, HR, Qr56, df	0.65	0.96	1.00	0.89	0.00	0.79	0.72	1.00	0.00	0.61	0.79	0.51	8.5	9
P, 120, HR, UB, none	0.24	0.83	0.51	0.44	1.00	1.00	0.78	0.05	1.00	0.78	0.89	0.82	10	10
N, 120, HR, Qr40, none	0.88	0.96	0.46	1.00	0.90	0.89	1.00	0.63	0.70	1.00	1.00	1.00	11	11

“Rank Clip” considers the rank sum of Clip 1, and “Final rank” is based on the mean of Clip 1/2 rank sum. For reconstruction parameters, see Table 1

*AV* Artefact volume, *CV* Clip volume, *df* Dental fillings, *FFT 1* Mean maximum amplitude of the low-frequency angular fast Fourier transform spectrum, *FFT 2* Mean integrated amplitude of the low-frequency fast Fourier transform spectrum, *HR* High-resolution mode, *Hr/Qr/UB* Manufacturer-specific reconstruction kernels, *MSSIM* Mean structural similarity index, *N* Siemens NAEOTOM Alpha, *nc* neuro coils, *P* Philips Brilliance Big Bore, *PIU* Percentage intensity uniformity, *PSNR* Peak signal-to-noise ratio, *Sn* Tin filtration, *STD* Standard deviation, *UHR* Ultra-high-resolution mode, *VMI* Virtual monoenergetic imaging, *X* Siemens SOMATOM X.ceed, *X/Y/Z* Clip widths

These results indicate that PCCT detector technology alone does not yield measurable improvements in artefact suppression compared to most recent EICT generations. The advantage of the PCCT becomes apparent primarily when compared with the older TP EICT platform (Fig. 6, right), where pronounced improvements in homogeneity and structural similarity were found.

In summary, the PCCT system achieved higher rating in quantitative performance in image uniformity PIU, STD, and PSNR, whereas the SOTA EICT performed slightly better for artefact-specific metrics such as AV, CV, and FFT 1/2.

### Subjective image evaluation

**Fig. 7:**
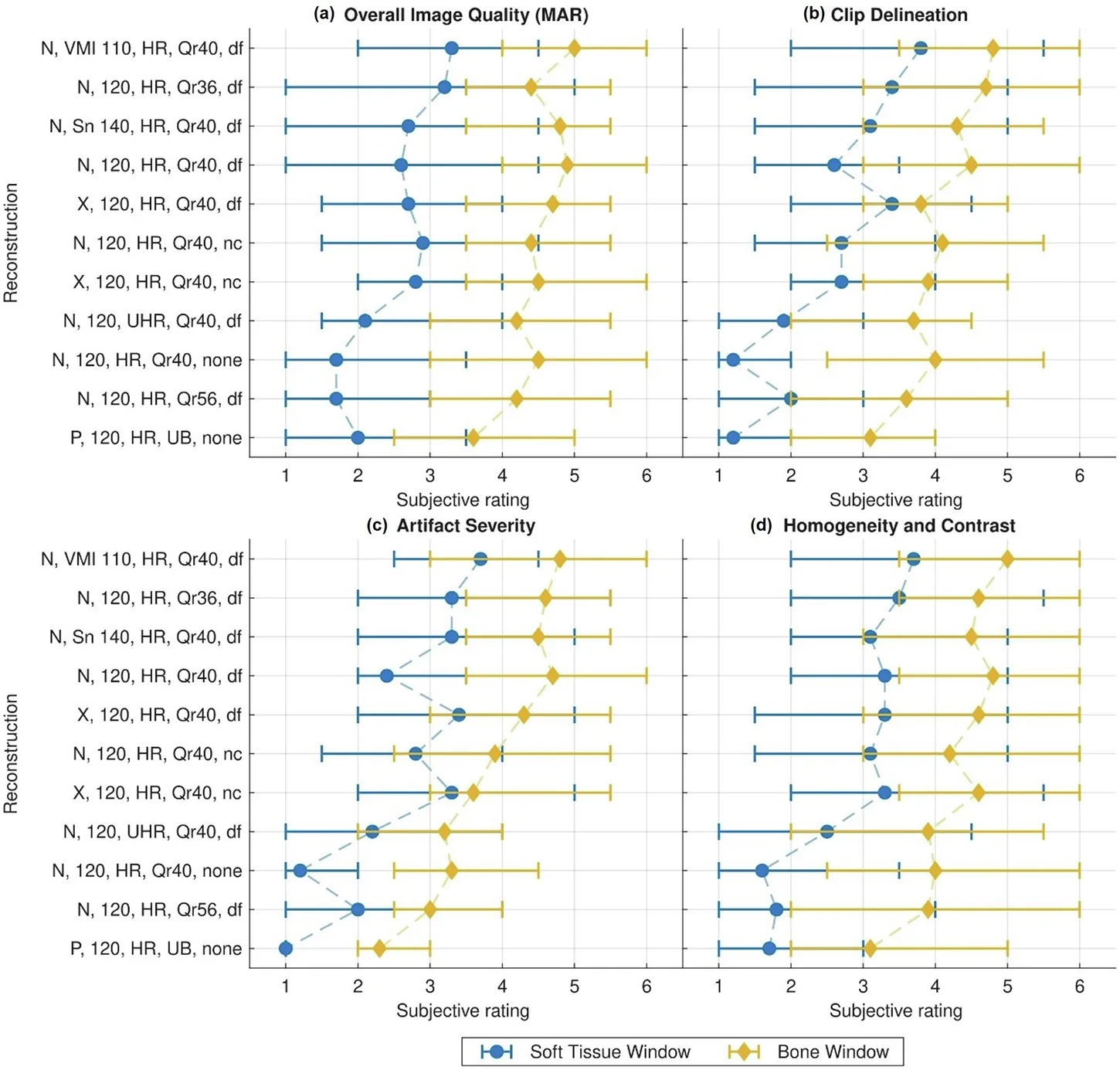
Mean subjective rating of (**a**) overall image quality (MAR), (**b**) clip delineation, (**c**) artifact severity and (**d**) homogeneity and contrast, based on the Likert scale (*e.g*., 1 = very poor image quality, 6 = excellent image quality), for the two phantoms and windows. The data points represent the average ratings from the subjective survey evaluations, while the error bars indicate the minimum and maximum rankings within the surveys. The dashed line serves as a visual guide. The reconstructions are listed from top to bottom, sorted by the overall image quality ranking (MAR). Detailed CT parameters for the displayed reconstructions can be found in Table 1. CT, Computer tomography; df, Dental fillings; HR, High-resolution mode; Hr/Qr/UB, Manufacturer-specific reconstruction kernels; MAR, Metal artefact reduction; N, Siemens NAEOTOM Alpha; nc, Neuro coils; P, Philips Brilliance Big Bore; Sn, Tin filtration; UHR, Ultra-high-resolution mode; VMI, Virtual monoenergetic imaging; X, Siemens SOMATOM X.ceed

A consistent trend in the ranking of reconstruction methods was observed across all evaluation categories, highlighting the robustness of the evaluation despite differing assessment criteria (Fig. 7).

The iMAR “dental fillings” algorithm was consistently rated higher to iMAR “neuro coils”, and PCCT was rated higher than the SOTA EICT. The monoenergetic PCCT reconstruction at 110 keV combined with iMAR “dental fillings” achieved the highest rating in all categories.

The Qr56 kernel received the lowest ratings, while Qr36 was rated highest. Sn 140 kV did not show a clear improvement over 120 kV, which was also used in the clinical protocol of the TP EICT and received the lowest overall rating. Reconstructions with smaller voxel sizes in the UHR setting were rated lower than those with larger voxel sizes. Overall, reconstructions in the bone window were rated better than those in the soft tissue window.

### Correlation of objective and subjective image evaluation

**Fig. 8:**
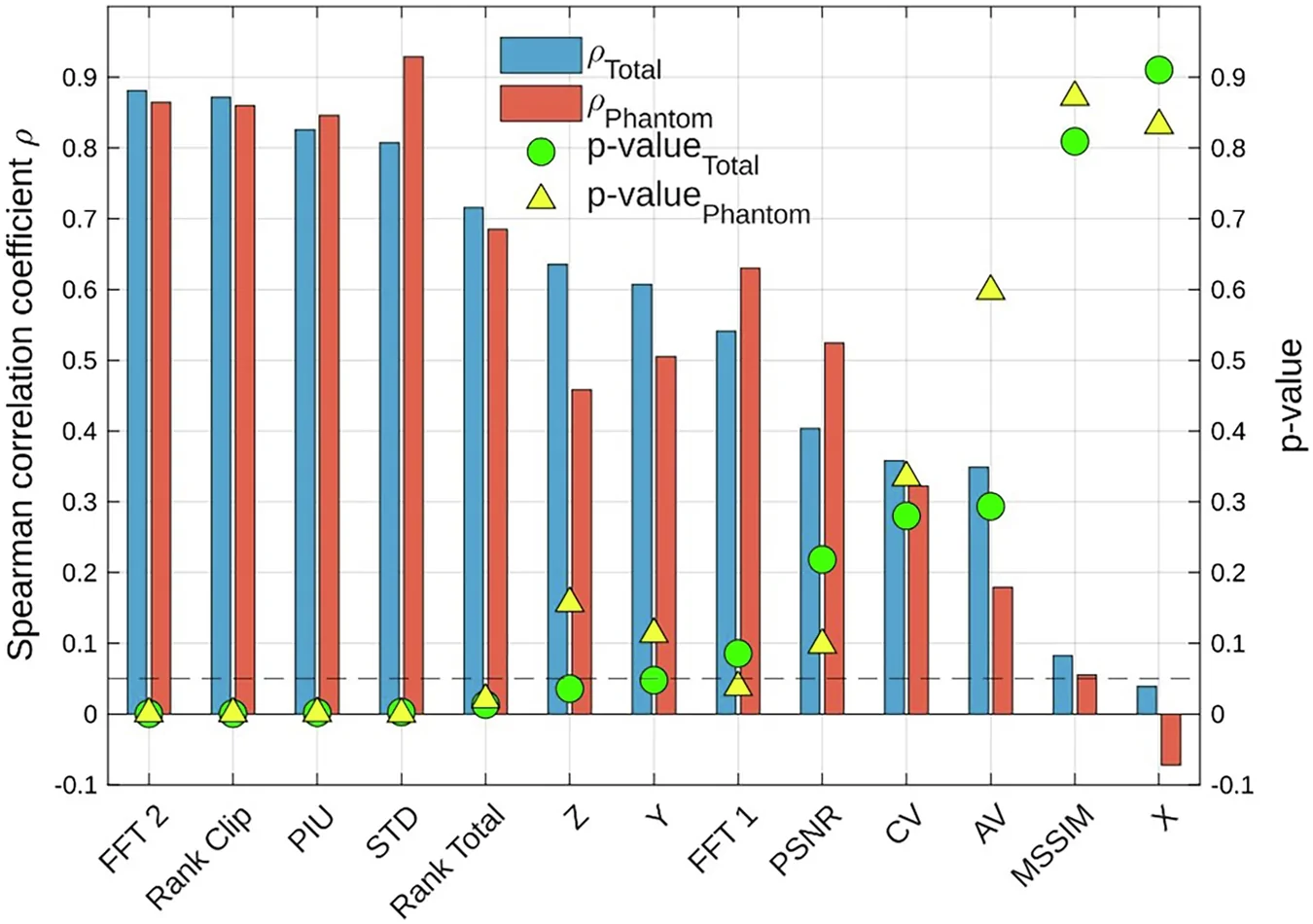
Spearman correlation coefficients (*ρ*_Total_ and *ρ*_Phantom_) and corresponding *p*-values between objective evaluation parameters and subjective image ratings (Phantom 1). The dashed horizontal line represents the statistical significance threshold (*p* = 0.05)

A strong agreement between the objective ranking scheme and the subjective image evaluation was found. Figure 8 summarises the Spearman correlation coefficients (*ρ*_*Total*_ and *ρ*_*Phantom*_), calculated between subjective and objective image quality evaluation for Phantom 1, with *Ρ*_*Total*_ reflecting the correlation with the global subjective ranking, whereas *ρ*_*Phantom*_ representing the correlation within phantom-specific evaluation. Comparable results were observed for Phantom 2 (not shown).

Parameters related to image homogeneity and artefact expression (PIU, STD, FFT 1, FFT 2) exhibited moderate to strong correlation with the subjective evaluation, confirming that the ranking-based objective evaluation reflects perceptual image quality well. In contrast, purely geometric metrics (X, Y, Z and CV) and global similarity measures (PSNR, MSSIM) showed weak or no correlation, *i.e*., being less representative of perceived diagnostic quality.

Both overall (Rank Total) and the clip-based ranking (Rank Clip) showed strong agreement with subjective perception, with *ρ*_Total_ = 0.76 (*p*_Total_ = 0.013) and *ρ*_Phantom_ = 0.69 (*p*_Phantom_ = 0.020) for overall ranking, and *ρ*_Total_ = 0.87 (*p*_Total_ = 0.001) and *ρ*_Phantom_ = 0.86 (*p*_Phantom_ = 0.001) for clip-based ranking (Phantom 1). For Phantom 2, *ρ*_Total_ = 0.81 (*p*_Total_ = 0.003) and *ρ*_Phantom_ = 0.86 (*p*_Phantom_ = 0.001) resulted for overall ranking, and *ρ*_Total_ = 0.77 (*p*_Total_ = 0.005) and *ρ*_Phantom_ = 0.82 (*p*_Phantom_ = 0.002) for clip-based ranking. The total results and the results across phantoms were comparable, underlining the objective ranking’s robustness approach. Notably, for Phantom 1, the higher ρ value for the clip-based ranking reflects the use of a single clip in the subjective analysis. The observed correlation coefficients indicate a strong and clinically meaningful association between objective metrics and reader preference, even though they did not reach the highest possible values (ρ ≥ 0.9). The associated *p*-values were below the 0.05 threshold, confirming that correlations were statistically significant [30].

## Discussion

This study systematically compared a clinical PCCT with conventional EICT for model-based OPT planning. PCCT offers a promising approach to enhance the visualisation of clips and surrounding structures. However, our findings indicate that these benefits are more evident in subjective image assessment than in objective quantitative metrics.

Tantalum clips challenge CT imaging due to their small size, high density and atomic number. The application of iMAR led to the most significant improvement in artefact reduction. To our knowledge, no manufacturer-specific iMAR configuration is tailored to tantalum, but “dental fillings” and “neuro coils” iMAR (Siemens) algorithms achieved the best results. These algorithms were originally designed for small metallic implants, typically composed of platinum-based alloys (*e.g*., neurovascular coils [31]), rather than high-Z materials. The reduction in artefacts resulted in a deterioration in low-contrast regions despite reduced noise, most likely due to blurring introduced [12, 32, 33]. Notably, the results of this study are specific to Siemens’ iMAR and may not match other manufacturers [12, 33].

VMI in PCCT without iMAR did not result in a significant reduction in artefacts in the objective analysis. Nevertheless, radiologists and radiation oncologist blinded to the scanner hardware preferred PCCT-VMI with iMAR in the subjective analysis in terms of overall image quality and clip delineation. The analysis of the VMI reconstructions between 65–110 keV alone showed no relevant artefact reduction. Improvement was only achieved by combining VMI at 110 keV with iMAR, with clear objective and subjective superiority in terms of image quality. As an isolated VMI option, 70 keV was favoured in the objective evaluation. While previous studies have primarily investigated high-energy VMI (> 100 keV) and implant materials such as titanium [34–36], tantalum exhibits different spectral characteristics due to its higher atomic number. A targeted investigation below the K-edge of tantalum (67.4 keV) in combination with iMAR could provide new approaches for material-dependent optimisation of image quality [7].

Increasing tube voltage with additional tin filtration slightly reduced metal artefacts, but caused losses in high and low contrast (up to 37%) and a potential increase in dose. Due to the low contrast of typical ocular lesions and structures, such an approach may impair tumour delineation accuracy.

With smaller voxel sizes (down to 0.09 × 0.09 × 0.1 mm³), both PCCT and EICT showed poorer results in artefact and contrast evaluation. This can be attributed to reduced signal per voxel and increased statistical noise, which amplify small intensity variations and streak patterns. The observed detrimental effect can be explained by the altered partial volume effect. While artefacts were in parts underestimated at low resolution, they appeared more differentiated in UHR mode, increasing their contribution to artefact metrics. Although not all possible combinations of reconstruction mode, matrix size, and FOV were investigated, the results show no relevant advantage of increased resolution for artefact-free reconstruction of tantalum clips.

Softer reconstruction kernels (Qr36/Hr36) were advantageous over the standard (Qr40/Hr40), while sharper kernels (Qr56/Hr56) resulted in poorer results. In addition, sharper kernels can lead to edge artefacts, thereby amplifying artificial transitions [37].

Despite similar objective performance of SOTA EICT, readers consistently rated the PCCT images as superior in their overall perception, indicating a clinically relevant subjective preference. The use of iMAR consistently improved perceived image quality and aligned well with the objective results. Although “neuro coils” achieved the most favourable objective artefact metrics, it also reduced low-contrast detectability, while “dental fillings” may provide a better trade-off between low-contrast detectability and artefact suppression, which may explain its consistently higher subjective ratings. Objective metrics such as PIU, STD and FFT-based amplitudes correlated moderately to strongly with the subjective assessment, whereas geometric measures, AV, PSNR and MSSIM showed weak or no correlation. These findings are in accordance with Große-Hokamp et al [25], supporting PIU and Fourier-analysis as suitable objective artefact metrics. Despite some interobserver variability, the ranking-based evaluation minimised bias. The multifaceted readers provided complementary perspectives by radiologists and radiation oncologists, supporting the clinical relevance of the results. Schmitt et al demonstrated improved MAR for tantalum-containing embolic agents using iMAR [38]. Their *in vitro* study on neurovascular coils showed that EICT provided superior MAR compared with PCCT without iMAR [39]. Aligning with our observations, combining PCCT with iMAR outperformed EICT [39]. The subjective preference for PCCT suggests that perception-relevant image characteristics (*e.g*., edge rendering or image texture) are not fully captured by the objective metrics used. Despite the higher dynamic range of the PCCT with 16 bits, the measured HU values for both phantoms and clip orientations (VMI 110 keV + iMAR dental fillings) lie within 10,857–24,008 HU [40]. Although the true HU value is not directly measurable, the theoretical HU for tantalum can be approximated as 374,171 HU based on its mass attenuation coefficient relative to water at diagnostic x-ray energies [41]. This highlights the significant challenge that tantalum presents for x-ray CT imaging, regardless of the use of iMAR and/or VMI reconstruction.

For proton therapy treatment planning, a conversion from HU to stopping-power ratios is required for dose calculation. The model-based approach in OPT typically overrides the registered, geometric eye model with a bulk material density [5]. With the significantly reduced artefacts and an adapted lookup table between HU and stopping-power ratios, a dose calculation on high-resolution PCCT may become feasible. A detailed investigation of range perturbations in the different reconstructions is left for future analysis. Improved clip delineation may reduce geometric uncertainties, contribute to treatment margins and support more accurate treatment planning, although this was not directly investigated [4, 5]. Alternative marker materials (*e.g*., titanium, carbon-based fiducials) have been investigated in the literature, but have not achieved widespread clinical adoption [42, 43].

Study limitations include CT system selection based on local availability and clinical relevance, with the Philips Brilliance Big Bore as a reference for older-generation EICT systems, rather than representing a comparison of all commercially available platforms. Future studies including other vendors and system generations could provide a more comprehensive evaluation. The comparison therefore reflects clinically scanner-reconstruction workflows rather than isolated detector performance, as only the Siemens systems used iMAR.

Our findings are consistent with the conclusions of the recent AAPM CT Metal Artefact Reduction Grand Challenge [44], which demonstrated a wide performance range among artefact reduction techniques and emphasised the dependence of artefact suppression on scanner hardware, algorithm design, and material type. The challenge further highlighted the persistent difficulty of reducing artefacts from high-Z materials, supporting our observation that tantalum clips remain particularly challenging for conventional artefact reduction approaches. The improved image homogeneity and clip delineation observed with PCCT, reflected by lower artefact volume and noise-related metrics as well as improved homogeneity measures, may demonstrate not only hardware-related advantages but also the potential of energy-resolved data for future MAR algorithm development and benchmarking. In future, deep-learning-based techniques may further improve imaging of high-density materials but were beyond the scope of the present study [44].

This study compared the image quality of tantalum clips in OPT using PCCT and two EICT systems. The objective analyses indicated only limited differences between PCCT and modern high-performance EICT systems, particularly regarding artefact reduction, with iMAR being the dominant factor for image enhancement. However, the blinded subjective evaluation revealed a significant preference for PCCT in terms of image quality and the delineation of tantalum clips. Combining iMAR with high-energy VMI achieved the largest reduction in artefact severity, while soft reconstruction kernels provided a favourable balance between image homogeneity and noise. These results underscore that PCCT offers relevant subjective advantages despite comparable objective results. The high degree of agreement among readers confirms the robustness of subjective perception and supports the quality of the findings. The study showed that higher resolution alone does not significantly improve image quality. In summary, PCCT is a promising technology for the subjective visualisation of implants and challenging materials such as tantalum in proton therapy. Further studies and algorithm development are required to clarify the clinical benefit and to optimise reconstruction strategies for this specific application.

## Supplementary information

**Additional file 1: Table S1:** Rank-based evaluation of objective image quality metrics of Phantom 1 for selected CT reconstructions from the Siemens NAEOTOM Alpha for evaluating the influence of the different CT parameters. **Table S2:** Rank-based evaluation of objective image quality metrics of Phantom 2 for selected CT reconstructions from the Siemens NAEOTOM Alpha for evaluating the influence of the different CT parameters. **Figure S1:** Objective evaluation of PCCT scans with and without iMAR. Deviation of the metrics for the iMAR “neuro coils”, “dental fillings”, and “extremity implants” compared to reconstruction without iMAR for Phantoms (a) 1 and (b) 2. Positive values indicate improvement and negative a degradation of respective metric (iMAR vs. no iMAR). Note: deviation >100 % are indicated at 100% level for better visibility (with actual values of 459 % (“dental fillings”), 150 % (“extremity implants”) and 416 % (“neuro coils”)).

## Data Availability

The datasets used and/or analysed during the current study are available from the corresponding author on reasonable request.

## Abbreviations

AV: Artefact volume
CNR: Contrast-to-noise ratio
CT: Computer tomography
CV: Clip volume
df: Dental fillings
EICT: Energy-integrating computer tomography
FFT: Fast Fourier transform
FFT 1: Mean maximum amplitude of the low-frequency angular fast Fourier transform spectrum
FFT 2: Mean integrated amplitude of the low-frequency fast Fourier transform spectrum
FOV: Field of view
HR: High-resolution
Hr/Qr/UB: Manufacturer-specific reconstruction kernels
HU: Hounsfield units
iMAR: Iterative metal artefact reduction
MAR: Metal artefact reduction
MSSIM: Mean structural similarity index
N: Siemens NAEOTOM Alpha
nc: Neuro coils
OPT: Ocular proton therapy
P: Philips Brilliance Big Bore
PA6: Polyamide 6
PCCT: Photon-counting computer tomography
PIU: Percentage intensity uniformity
PMMA: Polymethylmethacrylate
PSNR: Peak signal-to-noise ratio
PTFE: Polytetrafluorethylene
ROI: Region of interest
SOTA: State-of-the-art
STD: Standard deviation
TP: Treatment planning
UHR: Ultra-high-resolution
VMI: Virtual monoenergetic imaging
WC: Window centre
WW: Window width
X: Siemens SOMATOM X.ceed
X/Y/Z: Clip widths
