# Supplementary material for: Image quality and artefact reduction for tantalum clips in ocular proton therapy using photon-counting CT
Source: Eur Radiol Exp. 2026 Nov 13;10:183. doi: 10.1186/s41747-026-00819-7 (PMC13768496; doi:10.1186/s41747-026-00819-7)
Supplement: Supplementary file 1 [file 41747_2026_819_MOESM1_ESM.pdf]

Image quality and artefact reduction for tantalum clips in ocular proton therapy using photon-counting CT

ELECTRONIC SUPPLEMENTARY MATERIAL

Table S1: Rank-based evaluation of objective image quality metrics of Phantom 1 for selected CT reconstructions from the Siemens NAEOTOM Alpha for evaluating the influence of the different CT parameters. The average ranks of each metric describe the respective mean value of Clip 1 and 2 of the respective phantom.

**Varied CT parameter** (constant CT parameter)	average ranks											sum of rank			Final Rank
	X	Y	Z	CV	AV	PIU	STD	MSSIM	PSNR	FFT 1	FFT 2	Clip 1	Clip 2	total	
**iMAR** (U: 120 kV; CT mode: HR; matrix size: 512^2; FOV: 20 x 20 cm^2; kernel: Qr40; slice thickness: 0.4 mm; increment: 0.2 mm)															
neuro coils	0.50	0.50	0.50	0.14	0.00	0.07	0.07	0.43	0.07	0.07	0.07	0.09	0.00	0.00	1
extremity implants	0.50	0.50	0.50	0.00	0.50	0.07	0.07	0.57	0.07	0.21	0.07	0.00	0.30	0.11	2
dental fillings	0.50	0.50	0.50	0.64	0.21	0.43	0.29	0.14	0.36	0.21	0.29	0.33	0.31	0.29	3
hip implants	0.50	0.50	0.50	0.79	0.36	0.57	0.43	0.14	0.36	0.36	0.43	0.46	0.42	0.41	4
shoulder implant	0.50	0.50	0.50	0.82	0.71	0.29	0.71	0.57	0.71	0.71	0.71	0.56	0.74	0.63	5
pacemaker	0.50	0.50	0.50	0.75	0.50	0.79	0.64	0.86	0.64	0.64	0.64	0.71	0.61	0.64	6
thoracic coils	0.50	0.50	0.50	0.43	0.71	0.79	0.79	1.00	0.79	0.79	0.79	0.86	0.69	0.76	7
none	0.50	0.50	0.50	0.43	1.00	1.00	1.00	0.29	1.00	1.00	1.00	1.00	1.00	1.00	8
**VMI energy** (U: 120 kV; CT mode: HR; matrix size: 512^2; FOV: 20 x 20 cm^2; kernel: Qr40; slice thickness: 1 mm; increment: 0.7 mm; iMAR: dental fillings)															
70 keV	0.50	0.50	0.33	0.33	0.00	0.67	0.67	0.33	0.50	0.33	0.33	0.00	0.00	0.00	1
65 keV	0.50	0.50	0.33	0.00	0.33	0.33	0.33	1.00	1.00	0.00	0.00	0.33	0.44	0.40	2
110 keV	0.50	0.50	0.67	1.00	1.00	0.50	0.67	0.00	0.00	1.00	1.00	0.67	1.00	1.00	3
80 keV	0.50	0.50	0.67	0.67	0.67	0.50	0.33	0.67	0.50	0.67	0.67	1.00	0.94	0.86	4
**iMAR** (VMI energy: 110 keV; U: 120 kV; CT mode: HR; matrix size: 512^2; FOV: 20 x 20 cm^2; kernel: Qr40; slice thickness: 0.4 mm; increment: 0.4 mm)															
neuro coils	0.50	0.61	0.50	0.43	0.07	0.07	0.07	0.71	0.07	0.64	0.14	0.25	0.00	0.00	1
extremity implants	0.50	0.32	0.50	0.68	0.21	0.29	0.21	0.14	0.43	0.43	0.29	0.30	0.07	0.07	2
dental fillings	0.50	0.32	0.50	0.00	0.21	0.14	0.43	0.71	0.43	0.50	0.00	0.00	0.80	0.31	3
hip implants	0.50	0.32	0.50	0.57	0.36	0.57	0.36	0.29	0.57	0.43	0.43	0.50	0.35	0.35	4
shoulder implant	0.50	0.61	0.50	0.64	0.57	0.71	0.64	0.79	0.36	0.57	0.64	0.89	0.52	0.67	5
thoracic coils	0.50	0.61	0.50	0.89	0.86	0.57	0.64	0.57	0.50	0.36	0.71	0.75	0.79	0.74	6
shoulder implant	0.50	0.61	0.50	0.54	0.71	0.64	0.64	0.79	0.64	0.57	0.79	0.94	0.71	0.80	7
none	0.50	0.61	0.50	0.25	1.00	1.00	1.00	0.00	1.00	0.50	1.00	1.00	1.00	1.00	8
**Tube voltage** (CT mode: HR; matrix size: 512^2; FOV: 20 x 20 cm^2; kernel: Qr40; slice thickness: 0.4 mm; increment: 0.2 mm; iMAR: dental fillings)															
120 kV	0.50	0.50	0.50	0.00	1.00	1.00	0.50	0.00	0.50	0.00	0.00	0.00	1.00	0.50	1.5
Sn 140 kV	0.50	0.50	0.50	1.00	0.00	0.00	0.50	1.00	0.50	1.00	1.00	1.00	0.00	0.50	1.5
**Voxel size: CT mode, matrix size, FOV square size** (U = 120 kV; kernel: Qr40; slice thickness: 0.4 mm; increment: 0.2 mm; iMAR: dental fillings)															
HR, 1024^2, 400 cm^2	0.20	0.30	0.65	0.30	0.80	0.20	0.40	0.00	0.30	0.40	0.20	0.00	0.08	0.00	1
HR, 512^2, 400 cm^2	0.50	0.60	0.80	0.50	0.80	0.00	0.00	0.70	0.10	0.60	0.00	0.20	0.00	0.07	2
HR, 512^2, 81 cm^2	0.50	0.20	0.85	0.60	0.50	0.40	0.20	0.20	0.90	0.50	0.70	0.05	0.83	0.46	3
UHR, 1024^2, 400 cm^2	0.75	0.60	0.40	0.20	0.30	0.60	0.70	1.00	0.30	0.50	0.50	0.77	0.56	0.72	4
UHR, 512^2, 400 cm^2	0.65	0.30	0.15	1.00	0.30	1.00	1.00	0.50	0.50	0.40	0.80	1.00	0.78	0.97	5
UHR, 1024^2, 81 cm^2	0.40	1.00	0.15	0.40	0.30	0.80	0.70	0.60	0.90	0.60	0.80	0.82	1.00	1.00	6
**Kernel** (U: 120 kV; CT mode: HR; matrix size: 1024^2; FOV: 20 x 20 cm^2; slice thickness: 0.4 mm; increment: 0.2 mm; iMAR: dental fillings)															
Qr36	0.13	0.25	0.38	0.50	1.00	0.25	0.00	0.00	1.00	0.50	0.25	0.24	0.00	0.00	1
Qr40	0.50	0.25	0.38	0.00	0.50	0.25	0.50	0.50	0.50	0.50	0.25	0.00	0.38	0.07	2
Qr56	0.88	1.00	0.75	1.00	0.00	1.00	1.00	1.00	0.00	0.50	1.00	1.00	1.00	1.00	3
**Phantom Tilt – Clip** (U: 120 kV; CT mode: UHR; matrix size: 512^2; FOV: 20 x 20 cm^2; kernel: Qr40; slice thickness: 0.2 mm; increment: 0.1 mm; iMAR: dental fillings)															
0° - 2	0.83	1.00	1.00	0.67	0.00	0.00	0.00	1.00	0.33	0.00	0.00	-	-	0.00	1
45° - 1	0.17	0.33	0.33	0.00	0.33	0.67	0.67	0.33	0.67	0.33	0.33	-	-	0.44	2
0° - 1	0.83	0.33	0.00	1.00	1.00	0.33	0.33	0.67	0.00	1.00	1.00	-	-	0.72	3
45° - 2	0.17	0.33	0.00	0.33	0.67	1.00	1.00	0.00	1.00	0.67	0.67	-	-	1.00	4

AV, artefact volume; CT, computer tomography; CV, clip volume; FFT 1, mean maximum amplitude of the low-frequency angular fast Fourier transform spectrum; FFT 2, mean integrated amplitude of the low-frequency fast Fourier transform spectrum; FOV, field of view; HR, high-resolution mode; iMAR, iterative metal artefact reduction; MSSIM, mean structural similarity index; PIU, percentage intensity uniformity; PSNR, peak signal-to-noise ratio; Sn, tin filtration; STD, standard deviation; UHR, ultra-high-resolution mode; VMI, virtual monoenergetic imaging; X/Y/Z, clip widths

Table S2: Rank-based evaluation of objective image quality metrics of Phantom 2 for selected CT reconstructions from the Siemens NAEOTOM Alpha for evaluating the influence of the different CT parameters. The average ranks of each metric describe the respective mean value of Clip 1 and 2 of the respective phantom.

**Varied CT parameter** (constant CT parameter)	average ranks											sum of rank			Final Rank
	X	Y	Z	CV	AV	PIU	STD	MSSIM	PSNR	FFT 1	FFT 2	Clip 1	Clip 2	total	
**IMAR** (U: 120 kV; CT mode: HR; matrix size: 512^2; FOV: 20 x 20 cm^2; kernel: Qr40; slice thickness: 0.4 mm; increment: 0.2 mm)															
neuro coils	0.50	0.50	0.50	0.14	0.00	0.07	0.07	0.43	0.07	0.07	0.07	0.09	0.00	0.00	1
dental fillings	0.50	0.50	0.50	0.00	0.50	0.07	0.07	0.57	0.07	0.21	0.07	0.00	0.30	0.11	2
extremity implants	0.50	0.50	0.50	0.64	0.21	0.43	0.29	0.14	0.36	0.21	0.29	0.33	0.31	0.29	3
hip implants	0.50	0.50	0.50	0.79	0.36	0.57	0.43	0.14	0.36	0.36	0.43	0.46	0.42	0.41	4
thoracic coils	0.50	0.50	0.50	0.82	0.71	0.29	0.71	0.57	0.71	0.71	0.71	0.56	0.74	0.63	5
shoulder implant	0.50	0.50	0.50	0.75	0.50	0.79	0.64	0.86	0.64	0.64	0.64	0.71	0.61	0.64	6
pacemaker	0.50	0.50	0.50	0.43	0.71	0.79	0.79	1.00	0.79	0.79	0.79	0.86	0.69	0.76	7
none	0.50	0.50	0.50	0.43	1.00	1.00	1.00	0.29	1.00	1.00	1.00	1.00	1.00	1.00	8
**IMAR** (VMI energy: 110 keV; U: 120 kV; CT mode: HR; matrix size: 512^2; FOV: 20 x 20 cm^2; kernel: Qr40; slice thickness: 0.4 mm; increment: 0.4 mm)															
dental fillings	0.43	0.50	0.50	0.00	0.00	0.07	0.07	0.71	0.00	0.00	0.07	0.00	0.00	0.00	1
neuro coils	0.43	0.50	0.50	0.36	0.14	0.07	0.07	0.36	0.14	0.14	0.07	0.17	0.01	0.09	2
extremity implants	0.43	0.50	0.50	0.68	0.29	0.57	0.29	0.21	0.36	0.29	0.29	0.35	0.37	0.37	3
hip implants	1.00	0.50	0.50	0.96	0.43	0.57	0.43	0.29	0.36	0.43	0.43	0.50	0.45	0.48	4
shoulder implant	0.43	0.50	0.50	0.64	0.64	0.57	0.71	0.86	0.64	0.64	0.64	0.73	0.57	0.65	5
thoracic coils	0.43	0.50	0.50	0.71	0.71	0.57	0.71	0.36	0.64	0.71	0.71	0.55	0.83	0.69	6
pacemaker	0.43	0.50	0.50	0.32	0.79	0.57	0.71	0.86	0.86	0.79	0.79	0.87	0.60	0.74	7
none	0.43	0.50	0.50	0.32	1.00	1.00	1.00	0.36	1.00	1.00	1.00	1.00	1.00	1.00	8
**Tube voltage** (CT mode: HR; matrix size: 512^2; FOV: 20 x 20 cm^2; kernel: Qr40; slice thickness: 0.4 mm; increment: 0.2 mm; iMAR: dental fillings)															
Sn 140 kV	0.50	0.50	1.00	0.00	0.50	0.00	0.00	1.00	0.00	0.00	0.00	0.00	0.00	0.00	1
120 kV	0.50	0.50	0.00	1.00	0.50	1.00	1.00	0.00	1.00	1.00	1.00	1.00	1.00	1.00	2
**Voxel size: CT mode, matrix size, FOV square size** (U = 120 kV; kernel: Qr40; slice thickness: 0.4 mm; increment: 0.2 mm; iMAR: dental fillings)															
UHR, 512^2, 400 cm^2	0.50	1.00	0.33	0.33	0.17	0.33	0.50	0.67	0.17	0.50	0.67	0.82	0.00	0.00	1
HR, 1024^2, 81 cm^2	0.00	0.33	0.83	0.67	0.83	0.17	0.33	0.33	0.83	0.67	0.67	0.00	1.00	0.24	2
HR, 512^2, 400 cm^2	0.50	0.00	0.83	0.83	0.83	0.50	0.33	0.33	0.83	0.33	0.00	0.55	0.76	0.63	3
UHR, 1024^2, 81 cm^2	1.00	0.67	0.00	0.17	0.17	1.00	0.83	0.67	0.17	0.50	0.67	1.00	0.59	1.00	4
**Kernel** (U: 120 kV; CT mode: HR; matrix size: 512^2; FOV: 20 x 20 cm^2; slice thickness: 0.4 mm; increment: 0.2 mm; iMAR: dental fillings)															
Qr40	0.75	0.75	0.00	0.50	0.50	0.25	0.25	0.50	0.50	0.00	0.00	0.00	0.00	0.00	1
Qr36	0.00	0.00	0.75	0.00	1.00	0.25	0.25	0.00	1.00	0.75	0.50	1.00	0.41	0.75	2
Qr56	0.75	0.75	0.75	1.00	0.00	1.00	1.00	1.00	0.00	0.75	1.00	0.89	1.00	1.00	3

AV, artefact volume; CT, computer tomography; CV, clip volume; FFT 1, mean maximum amplitude of the low-frequency angular fast Fourier transform spectrum; FFT 2, mean integrated amplitude of the low-frequency fast Fourier transform spectrum; FOV, field of view; HR, high-resolution mode; iMAR, iterative metal artefact reduction; MSSIM, mean structural similarity index; PIU, percentage intensity uniformity; PSNR, peak signal-to-noise ratio; Sn, tin filtration; STD, standard deviation; UHR, ultra-high-resolution mode; VMI, virtual monoenergetic imaging; X/Y/Z, clip widths

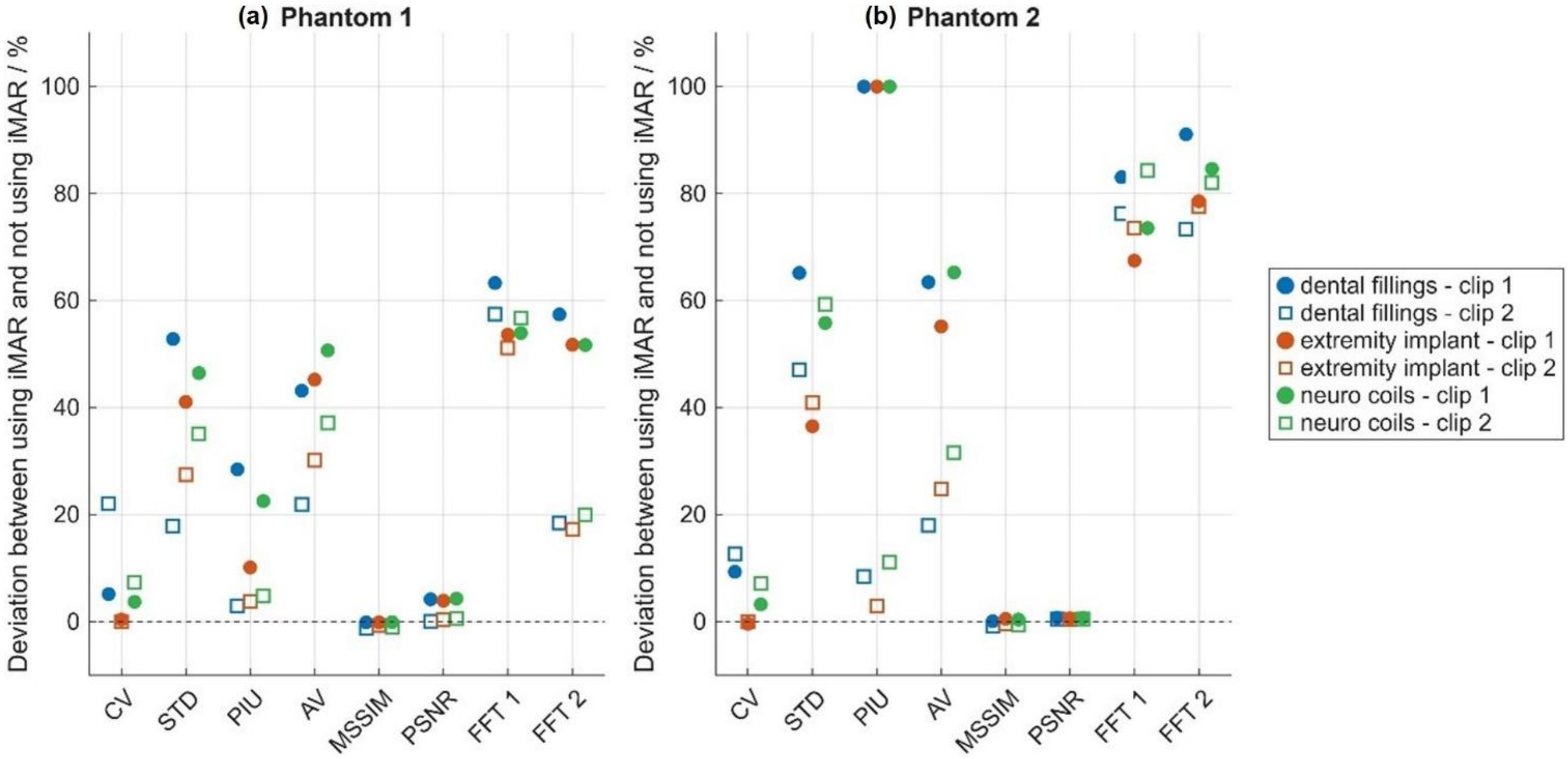

Figure S1: Objective evaluation of PCCT scans with and without iMAR. Deviation of the metrics for the iMAR “neuro coils”, “dental fillings”, and “extremity implants” compared to reconstruction without iMAR for Phantoms (a) 1 and (b) 2. Positive values indicate improvement and negative a degradation of respective metric (iMAR vs. no iMAR). Note: deviation >100 % are indicated at 100% level for better visibility (with actual values of 459 % (“dental fillings”), 150 % (“extremity implants”) and 416 % (“neuro coils”)). iMAR, iterative metal artefact reduction; PCCT, photon-counting computer tomography
